# Impact of Long-Term Hippotherapy on the Walking Ability of Children With Cerebral Palsy and Quality of Life of Their Caregivers

**DOI:** 10.3389/fneur.2019.00834

**Published:** 2019-08-13

**Authors:** Tomoko Mutoh, Tatsushi Mutoh, Hirokazu Tsubone, Makoto Takada, Misato Doumura, Masayo Ihara, Hideo Shimomura, Yasuyuki Taki, Masahiro Ihara

**Affiliations:** ^1^Department of Nuclear Medicine and Radiology, Institute of Development, Aging and Cancer, Tohoku University, Sendai, Japan; ^2^Division of Clinical Psychology, Graduate School of Human Sciences, Kobe Shoin Women's University, Kobe, Japan; ^3^Department of Veterinary Medical Sciences, Graduate School of Agricultural and Life Sciences, The University of Tokyo, Tokyo, Japan; ^4^Life Science Institute, Inc., Tokyo, Japan; ^5^Holistic Betterment and Wellness Through Riding PIROUETTE, Utsunomiya, Japan; ^6^Faculty of Medical Technology, Teikyo University, Utsunomiya, Japan

**Keywords:** hippotherapy, cerebral palsy, gait analysis, caregivers, quality of life

## Abstract

**Background:** Cerebral palsy (CP) is a permanent motor disorder that occurs at birth or during early infancy. Despite advances in fetal and maternal medicine, the incidence of CP remains high. Hippotherapy has gradually been recognized as an excellent rehabilitation tool for children with CP. However, a scientific basis for how it achieves long-term functional improvements or provides additional benefits to patients' caregivers remains unknown.

**Objectives:** We performed a prospective trial to determine how hippotherapy affects the gross motor and gait functions in children with CP and how it may also impact the quality of life (QOL) of patients' caregivers.

**Methods:** In total, 24 children with CP (11 boys, 13 girls; age: 4–14 years; Gross Motor Function Classification System [GMFCS] II-III) underwent a program (30 min/day, once a week) of hippotherapy or day-care recreation (control) over a 1-year intervention and a 3-month follow-up period. Assessment measures used for the children were gait parameters for a 5-m walk test, Gross Motor Function Measure (GMFM)-66, and GMFM dimension-E (GMFM-E). The QOL of the caregivers was estimated using a brief version of the World Health Organization Quality Of Life (WHOQOL-BREF) self-assessment questionnaire.

**Results:** In addition to better GMFM-66 and GMFM-E scores, hippotherapy was associated with increased cadence, step length, and mean acceleration; stabilized horizontal/vertical displacement of patients; and better relationship between the psychological status and QOL of the caregivers than those seen in the control group (*p* < 0.05). Additionally, the initially improved children's step length and their caregivers' psychological QOL domain (particularly in the “positive feeling” facet) tended to be preserved up to the 3-month follow-up.

**Conclusion:** These data suggest that compared with common day-care recreational activities, a 1-year program of once-weekly hippotherapy can improve not only the walking ability of children with CP but also the psychological health and QOL of their caregivers.

**Clinical Trial Registration:**: www.umin.ac.jp/ctr/, identifier: UMIN000022986

## Introduction

Cerebral palsy (CP) is a permanent motor disorder caused by non-progressive encephalopathies of varied etiologies at birth or during early infancy, which are strongly associated with public health issues (1). Despite advances in fetal and maternal medicine, the incidence of CP is still at 2–2.5/1,000 live births (2). The actual performance in daily life of children with CP has been shown to be affected not only by their physical environment but also by social and psychological factors.

Hippotherapy (*hippo* from the Greek for horse) is a popular animal-assisted therapy that utilizes equine movements as part of a comprehensive program of intervention aimed at improving functional outcomes because it enhances the muscle tone, balance, and coordination through horseback riding (3–5). Hippotherapy has gradually been recognized as a potential tool for rehabilitation in children with CP as part of their broader integrated plan of care (6). However, recent meta-analyses and systematic reviews show that there is still insufficient evidence to support the long-term benefits of hippotherapy for the total Gross Motor Function Measure (GMFM) or quality of life (QOL) parameters (7–10), although some improvements in postural alignment and balance of the head and trunk and lower limb muscle activity have been found after short-term hippotherapy (typically involving 20–30-min treatments once or twice a week for up to 25 consecutive weeks) (3, 11–17). Additionally, several of our recent studies have shown that over a 1-year hippotherapy program, the step length and average acceleration at 6 weeks suggested an improvement in the GMFM scores of children and adolescents with CP (18, 19).

In terms of psychosocial aspects of children with CP, it is being increasingly accepted that the best approach is family-centered care that requires the primary caregiver, usually the mother, to be an active participant in their child's care. However, most caregivers tend to have a poor QOL due to a sense of inferiority or guilt, as they experience serious impacts on the time available for normal life tasks and spending with non-disabled children, and they often have a sense of being isolated by their neighbors (20). Unfortunately, hippotherapy currently lacks a scientific basis for how it improves the QOL of these caregivers. Several earlier reports have suggested that hippotherapy motivates children to actively participate in social activities (5, 21); however, no psychological or emotional improvements in the QOL of mothers of children with CP have been demonstrated in small observational studies (22, 23).

We hypothesized that if we could use a more formal and rigorous approach of hippotherapy, further positive effects on children with CP and on the QOL of their caregivers would be observed. Such studies would employ a design using control subjects of a similar sample size and characteristics, with sufficient intensity and duration of therapy to achieve physical improvements and ability to perform fun activities.

Therefore, to test this hypothesis, we conducted a prospective clinical trial to examine not only the effects of hippotherapy on functional and balance improvements in children with CP but also its ancillary effects that may impact the QOL of their primary caregivers.

## MethodS

### Participants and Setting

All eligible participants were recruited and tested from July 2016 to March 2018. Children with CP were recruited prospectively by contacting the schools that cater to such children by providing appropriate physiotherapy services and via adjacent university and/or other general hospitals. A total of 30 participants were enrolled. All participants had (1) the ability to walk with or without aids or braces, (2) a prior diagnosis of spastic CP, and (3) an ability to train for more than 30 min. There were four exclusion criteria: (1) having had any orthopedic surgery or other surgical interventions that may influence mobility in the past 6 months, (2) having received a botulinum toxin injection in the past 3 months or a selective dorsal rhizotomy in the past 1 year, (3) impairments that could contraindicate horseback riding, and (4) other medical conditions that could materially influence mobility. No participants had any previous experience with hippotherapy.

### Design

This study was designed as a randomized controlled trial. Participants were allocated by computer-generated stratified randomization according to the severity of their impairment (assessed by the Gross Motor Function Classification System [GMFCS]) to one of the following two treatment groups: outdoor recreation (control) and hippotherapy. BellCurve for Excel software (SSRI, Tokyo, Japan) was used to calculate the appropriate sample size. Based on the results of a previous cohort study (24), 11 children in each group were needed to detect a mean change of 3% in the Gross Motor Function Measure (GMFM)-66 scores in order to benefit from hippotherapy (with the assumption of no apparent change detected in the control group receiving conventional physiotherapy) with a power of 80% and type-I error rate of 0.05.

### Ethics Approval and Registration

This study was conducted in compliance with the ethical guidelines of the Declaration of Helsinki and was approved by the Human Research Ethics Committee of the Society of Physical Therapy Science (SPTS2016007). All experiments in this study were performed in accordance with the relevant guidelines and regulations, and written informed consent was obtained from the caregivers of all study subjects. Permission to use the animals was waived because the owners of the animals were part of the research team. The trial was registered in the University hospital Medical Information Network Clinical Trials Registry (UMIN-CTR) on 2 July 2016 (UMIN000022986).

### Protocol

Hippotherapy was performed at the Holistic Betterment and Wellness Through Riding PIROUETTE riding center (http://www.pirouet-u.com/) formerly known as Riding for the Disabled Association (RDA) Utsunomiya (Tochigi Prefecture), located in the Kanto region–Japan's largest plain consisting of seven prefectures including the capital city of Tokyo. The facility has licensed and specially trained members including certificated therapeutic riding instructors, occupational therapists, psychologists, medical doctors, and veterinarians and conducts highly dedicated educational and research activities on hippotherapy (18, 19, 25, 26).

Participants received a 30-min program of hippotherapy once a week for 48 consecutive weeks (1 year) in an outdoor riding arena. Horses and participants were matched according to the size and functional status of the children with the size and movement characteristics of specific horses, as best as possible. The hippotherapy used in this study was based on the standard program (21), which included muscle relaxation and sustenance of optimal postural alignment of the head, trunk, and lower extremities with independent sitting and active exercises (stretching, strengthening, dynamic balance, and postural control), as directed by the instructor (Figure S1A). In contrast, participants assigned to the control group received a weekly recreation program using outdoor amenities such as green spaces, walking path, and playground equipment using the same time schedule as the hippotherapy cohort (i.e., a 1-year weekly program followed by a 3-month follow-up). All participants continued their normal daily routines for the rest of the week, but none of them received routine physical therapy during the study period.

In both groups, caregivers were allowed to watch the session without participating in the protocol or discussing the contents or answers of the self-reported questionnaire form with each other so as to avoid bias via the formation of alliances with other caregivers during the session, which could have contributed to a feeling of psychological well-being, especially in the hippotherapy caregiver group.

### Measurements

Physical function was assessed using the 5-m walk test (5MWT) (27) before and after each session at self-selected walking speeds (18). The 5MWT was performed on a 7-m walkway, and the walk in the middle 5 m of the walkway was timed (Figure S1B). Participants who habitually wore ankle–foot orthoses and/or used a walking aid were permitted to use these for the test. Temporospatial analyses of the gait and balance abilities were performed using a portable motion recorder (MG-M1110-HW; LSI Medience, Tokyo, Japan) (Figure S2). The resulting motion signals were then recorded at a sampling rate of 100 Hz, stored on a computer for off-line analysis (MG-M1110-PC; LSI Medience, Tokyo, Japan), and then used to quantify cadence (step/min), walking speed (m/min), step length (cm), average acceleration (G), and the horizontal (side-to-side motion of the pelvis) and vertical (rise and fall of the center of gravity) displacement ratio (18, 19, 26).

The overall QOL of caregivers was investigated using the brief version of the World Health Organization Quality Of Life (WHOQOL-BREF) self-assessment questionnaire derived from the WHOQOL-100 (28). It contains 26 universal items addressing five domains of the QOL: (1) general health (two items), (2) physical health (seven items), (3) psychological health (six items), (4) social relationships (three items), and (5) environment (eight items) (https://www.who.int/healthinfo/survey/WHOQOL_BREF.pdf?ua=1). These domains and terms were agreed upon by the international consensus and adapted into a Japanese version (29). In this study, the WHOQOL-BREF was self-administered since all of the respondents were considered to have sufficient ability to complete the form, as described (30). The domain scores were scaled in a positive direction ranging from 1–5 points, with higher scores denoting better QOL.

### Outcome Measures

Temporospatial gait parameters and QOL domains of the caregivers were compared between the two groups before (baseline) and after the 1-year intervention and at the follow-up 3 months after completion of the training (post-training). GMFM-66 and GMFM dimension-E (GMFM-E) (walking, running, and jumping) scores were measured for outcome analyses by the same blinded examiner. Each measurement was performed under the same conditions for a total of three times: the first one was made the week before beginning the intervention, the second one after the completion of the 1-year intervention, and the final one on the day of the 3-month follow-up.

### Statistical Analysis

Continuous data that were normally distributed according to the D'Agostino–Pearson normality test were compared using analysis of variance (ANOVA) with *post hoc* Bonferroni–Dunn correction. Non-normally distributed variables were assessed using the Mann–Whitney *U* test. Univariate analyses of relationships between the categorical variables and outcomes of interest were performed using the χ^2^ test or Cochran–Armitage test as appropriate. Linear regression analysis was used to compare the average acceleration or horizontal/vertical displacement ratio with cadence (potential predictors of gait and balance stabilities for long-term hippotherapy) (19) for within-group correlation both before and after the intervention. Multinomial logistic regressions were fitted to determine if data plots for each group were associated with the referenced normal database (NDB) values predefined using age-matched healthy subjects (Figure S3). All analyses were performed using SPSS (version 24.0; IBM, Armonk, NY, USA) and Prism (version 8; GraphPad Software, La Jolla, CA, USA). Data are expressed as mean ± standard deviation (SD). The level of significance was set at *p* < 0.05.

## Results

### Study Population and Outcomes

**Figure 1:**
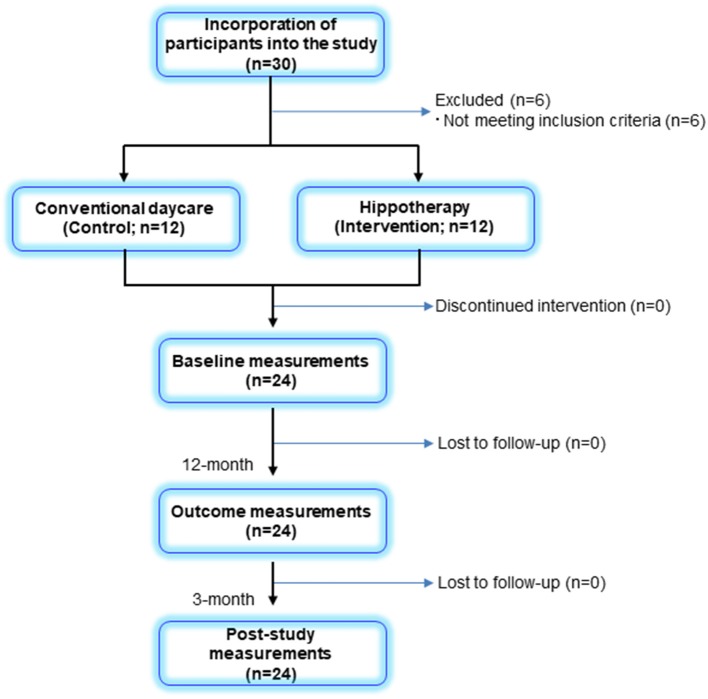
Flow diagram of participants in the study.

Among the 30 participants enrolled in this study, a total of 24 children and their caregivers completed both interventions and were included in the analysis (Figure 1). Descriptive statistics of the participants in each group are shown in Table 1. Overall, the mean age of children was 9 ± 3 years (range, 4–14 years), and all children had bilateral spastic CP of the lower limbs. Further, 8 children in the GMFCS III (57%) group used ankle–foot orthoses bilaterally but were sufficiently ambulatory to successfully execute a 5MWT. All participants completed the 48 sessions of hippotherapy or the day-care recreation program for over 1 year and were subsequently followed up with a mean post-intervention time of 12.8 ± 0.9 weeks (range, 12–15 weeks). No adverse events were reported throughout the study.

**Table 1:** Participants' characteristics.

	**Hippotherapy (*n* = 12)**	**Control (*n* = 12)**	***p*-value**
**Children**			
Age (years)	8 ± 3 (4–14)	9 ± 3 (7–14)	0.43
Sex (male/female)	5/7 (42/58)	6/6 (50/50)	0.26
**GMFCS levels**			
II	5 (42)	5 (42)	1.00
III	7 (58)	7 (58)	
Post-intervention follow-up duration (weeks)	12.8 ± 0.9 (12–14)	13.1 ± 1.0 (12–15)	0.43
**Primary caregivers**			
Age (years)	38 ± 6 (28–47)	36 ± 6 (27–45)	0.44
Respondent (mother/father)	11/1 (92/8)	12/0 (100/0)	0.31
**Educational status**			
College (2-year degree program)	1 (8)	2 (16)	0.54
University (4-year or more)	11 (92)	10 (84)	

*Data are expressed as mean ± SD (range) or number (percentage). GMFCS, gross motor function classification system*.

Table 2 shows the mean GMFM-66 and GMFM-E scores together with their corresponding SDs at the three aforementioned time points. Baseline scores for each outcome estimate were not significantly different between the two groups. The 1-year hippotherapy program significantly improved children's GMFM scores (*p* = 0.027 for GMFM-66 and *p* = 0.044 for GMFM-E) compared with the scores in the control group. Although increased GMFM-66 and GMFM-E scores from the baseline persisted till the 3-month follow-up in the hippotherapy group (*p* = 0.029 for GMFM-66 and *p* = 0.027 for GMFM-E; within-group interaction), statistically significant differences between the two interventions could be detected only for the GMFM-E score (*p* = 0.039).

**Table 2:** Outcome measures before and after the intervention in children with CP.

	**Baseline**	**1-year**	**Follow-Up**
**GMFM-66 (%)**			
Control	57.4 ± 7.9	57.9 ± 9.2	58.2 ± 7.5
Hippotherapy	56.6 ± 9.2	62.8 ± 10.8*†	61.1 ± 9.5*
**GMFM-E (%)**			
Control	46.0 ± 6.3	46.5 ± 6.6	45.8 ± 5.5
Hippotherapy	45.4 ± 7.0	49.7 ± 7.6*†	48.5 ± 5.3*†
**Cadence (step/min)**			
Control	86.4 ± 19.2	87.8 ± 16.4	92.3 ± 16.2
Hippotherapy	79.3 ± 28.8	104.4 ± 24.3*†	98.3 ± 18.4*
**Walking speed (m/min)**			
Control	31.1 ± 11.3	32.3 ± 11.6	31.1 ± 11.8
Hippotherapy	31.9 ± 10.7	38.8 ± 13.5*†	36.6 ± 11.8
**Step length (cm)**			
Control	29.8 ± 6.9	30.9 ± 7.7	30.4 ± 8.3
Hippotherapy	33.3 ± 10.1	36.8 ± 8.4*†	35.4 ± 9.1*†
**Average acceleration (G)**			
Control	0.31 ± 0.09	0.32 ± 0.08	0.32 ± 0.08
Hippotherapy	0.29 ± 0.09	0.38 ± 0.12*†	0.38 ± 0.10*
**H/V displacement ratio**			
Control	3.72 ± 1.65	3.55 ± 1.53	3.39 ± 1.49
Hippotherapy	3.89 ± 1.95	2.61 ± 1.00*†	2.69 ± 1.22*

*Values are expressed as mean ± SD. GMFM, gross motor function measure; GMFM-E, walking, running, and jumping elements of the GMFM-88; H/V, horizontal/vertical*.

* *p <0.05 vs. Baseline*;

† *p <0.05 vs. Control*.

### Time-Course Changes of Functional Gait Parameters

Over the course of the study period, there was a significant increase in cadence, walking speed, step length and average acceleration (*p* < 0.001; within-group time effect) during the 5MWT in participants receiving hippotherapy (Table 2). A decrease in the horizontal/vertical displacement ratio was also noted (*p* = 0.009). Statistically significant differences in these gait parameters were demonstrated between the two groups after 1-year of intervention (*p* < 0.01), and the increased step length was further maintained at the 3-month follow-up after hippotherapy (*p* = 0.018).

### Comparison With Normal Gait Functions

**Figure 2:**
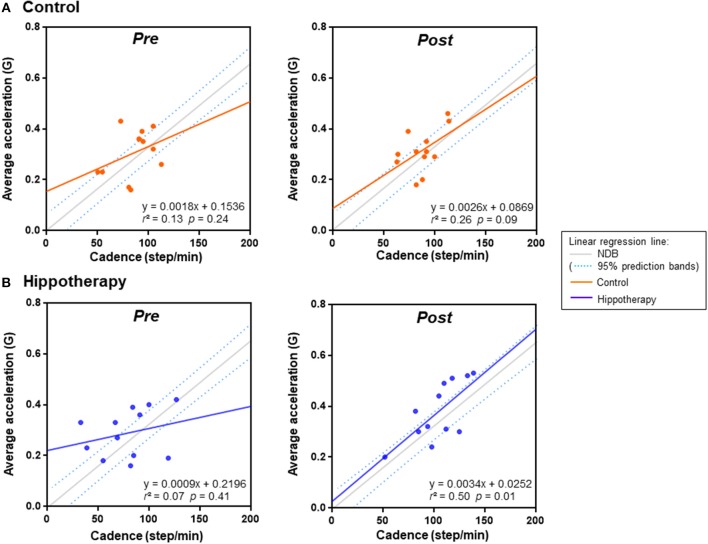
Linear regression analysis of average acceleration and cadence in each group before (pre) and after (post) the 1-year intervention. **(A)** control group, **(B)** hippotherapy. Dashed lines indicate 90% prediction intervals of the reference regression line in the age-matched normal database (NDB; Figure S3 for details).

To examine the relationship of gait (cadence) and balance (horizontal/vertical displacement ratio) with gait strength (average acceleration) during 5MWT, we ran a simple linear regression model for each intervention compared with an age-matched NDB (*n* = 50). The scatterplot of the gait cadence and average acceleration in the post-hippotherapy group was linear (*r* = 0.71; *p* = 0.01) and tended to be close to that of the NDB value (*F* = 11.312; *p* = 0.023), whereas that before hippotherapy or in the control groups was not linear (*r* = 0.26–0.51; *p* ≥ 0.05) (Figure 2).

**Figure 3:**
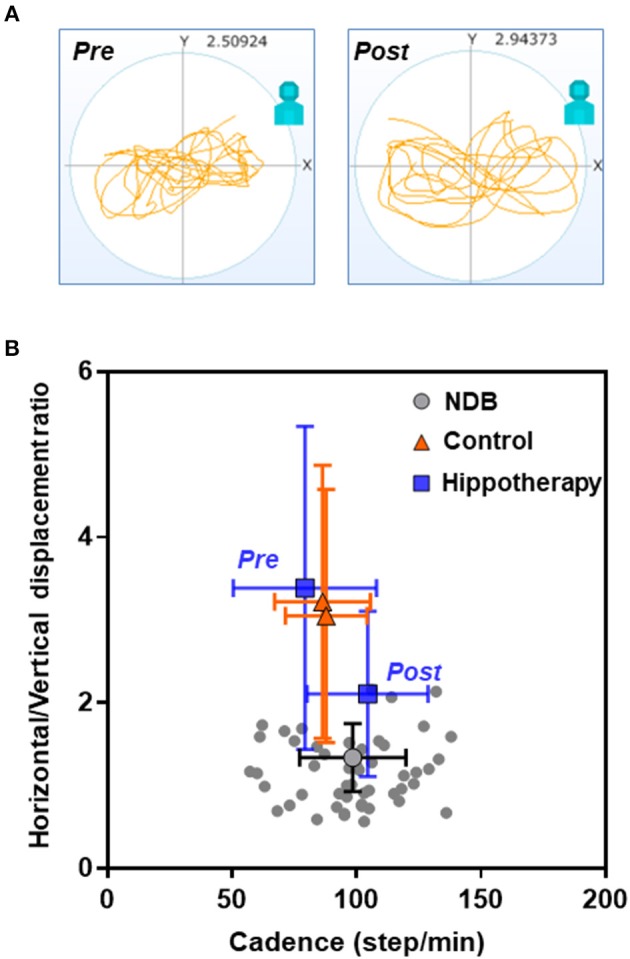
Relationships of cadence and horizontal/vertical displacement ratio in each group before and after the 1-year intervention. **(A)** Representative data from a child with CP before (pre) and after (post) hippotherapy; **(B)** Mean plots with both cadence and horizontal and vertical standard deviations. Note that there is an apparent shift in the mean ± SD plot in the hippotherapy group (blue square) toward the plots in the age-matched normal database (NDB; Figure S3 for details) (gray circle) after the intervention.

Regarding the relationship between the horizontal/vertical displacement ratio and average acceleration, there was an apparent shift in the bidirectional mean ± SD plot toward the area of plots in the age-matched NDB after the intervention in the hippotherapy group (*F* = 38.16; *p* < 0.001) (Figure 3). Further, the results of simple linear regression analysis indicated a statistically significant relationship between the post-hippotherapy group and NDB (α [95% confidence interval] = −5.097 [−8.894 to −1.301]; *p* = 0.009).

### Caregiver QOL

For the WHOQOL-BREF score, no significant differences were reported in 4 QOL domains of scores across general, physical, social, and environmental health (*p* > 0.05), but positive effects were observed on psychological health immediately after hippotherapy, and at the 3-month follow-up, as compared to the control group (*p* < 0.05) (Table 3). In subgroup analyses among individual facets of the psychological domain, caregivers had higher scores on the “positive feeling” and “self-esteem” items after hippotherapy (*p* < 0.01), and the former tended to be preserved at the follow-up (*p* < 0.05) (Table 4).

**Table 3:** Caregiver functions according to the QOL domains of the WHOQOL-BREF before and after the intervention in children with CP.

**Domains (score range)**	**Baseline**	**1-year**	**Follow-Up**
**General health (2–10)**			
Control	6.3 ± 1.2	6.3 ± 1.1	6.2 ± 1.3
Hippotherapy	6.2 ± 1.3	6.6 ± 1.2	6.5 ± 1.0
**Physical (7–35)**			
Control	22.7 ± 5.1	23.2 ± 4.2	22.5 ± 4.8
Hippotherapy	21.7 ± 4.7	23.8 ± 4.9	22.8 ± 5.6
**Psychological (6–30)**			
Control	18.0 ± 3.7	18.2 ± 3.3	17.8 ± 3.0
Hippotherapy	17.8 ± 4.9	20.4 ± 4.3*†	19.8 ± 4.6*†
**Social (3–15)**			
Control	10.3 ± 2.9	10.4 ± 3.1	10.3 ± 3.1
Hippotherapy	10.5 ± 2.2	10.8 ± 2.2	10.8 ± 2.2
**Environmental (8–40)**			
Control	25.3 ± 8.9	25.5 ± 8.1	25.4 ± 8.3
Hippotherapy	23.3 ± 7.2	23.3 ± 7.0	23.4 ± 6.8

*Values are expressed as mean ± SD*.

*WHOQOL-BREF, brief version of the World Health Organization Quality of Life questionnaire*.

* *p <0.05 vs. Baseline*;

† *p <0.05 vs. Control*.

**Table 4:** Individual facets of the psychological domain of the WHOQOL-BREF before and after the intervention in children with CP.

**Facets (6 items)**	**Baseline**	**1-year**	**Follow-Up**
**Positive feelings**			
Control	3.1 ± 0.9	3.4 ± 1.0	3.3 ± 0.8
Hippotherapy	3.1 ± 1.0	4.1 ± 1.0*†	3.8 ± 0.7*†
**Spirituality/religion/personal beliefs**			
Control	2.6 ± 0.8	2.6 ± 0.7	2.8 ± 0.8
Hippotherapy	2.7 ± 1.1	2.7 ± 1.1	2.8 ± 1.1
**Thinking/learning/memory/concentration**			
Control	2.8 ± 0.9	2.9 ± 0.8	2.9 ± 0.7
Hippotherapy	3.1 ± 0.9	3.2 ± 0.9	3.3 ± 0.9
**Bodily image and appearance**			
Control	3.3 ± 0.8	3.1 ± 0.8	3.1 ± 0.6
Hippotherapy	3.1 ± 1.1	3.6 ± 1.0	3.5 ± 1.0
**Self-esteem**			
Control	3.3 ± 1.1	3.4 ± 0.7	3.3 ± 0.8
Hippotherapy	2.9 ± 1.2	4.0 ± 0.7*†	3.5 ± 1.1*
**Negative feelings**			
Control	2.8 ± 0.8	2.8 ± 0.8	2.5 ± 0.7
Hippotherapy	2.9 ± 0.8	2.8 ± 0.7	2.8 ± 0.9

*Values are expressed as mean ± SD*.

WHOQOL-BREF, brief version of the World Health Organization Quality of Life questionnaire.

* *p <0.05 vs. Baseline*;

† *p <0.05 vs. Control*.

**Figure 4:**
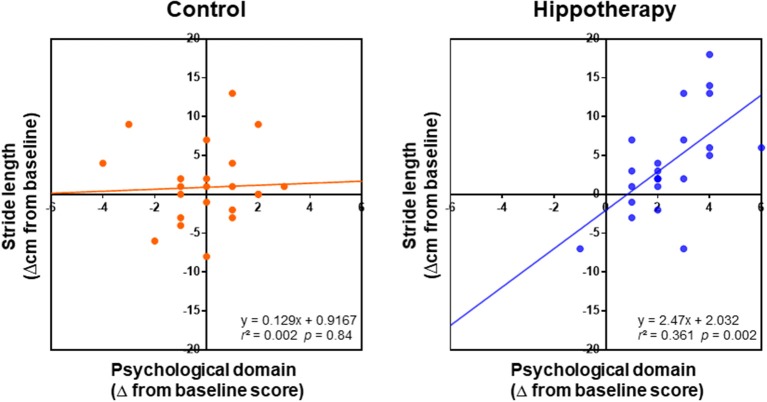
Relationships between changes in the psychological domain of caregivers and children's step length (Δ from baseline) in each group after the intervention; grouped data for 1-year intervention and 3-month follow-up were analyzed using simple linear regression analysis. Note that the regression shows a positive relationship between the QOL of caregivers and children's step length in the scatterplots of the hippotherapy group after the intervention.

With regard to the relationship between caregiver QOL and the gait function of children, linear regression analysis showed a positive relationship between step length increase and improvements in psychological domain of the caregivers (Δ from the baseline) post-hippotherapy up to the 3-month follow-up (*r* = 0.61; *p* = 0.002), but not in the control group (*r* = 0.04; *p* = 0.84) (Figure 4). In the hippotherapy group, the step length also had a significant correlation with one item, i.e., “positive feeling” within the psychological QOL domain (*r* = 0.54; *p* = 0.006). No other items in both groups revealed any significant correlation coefficients (*p* ≥ 0.05).

## Discussion

Mobility is one of the most common issues associated with CP, and therefore, therapies that increase lower body mobility, an important aspect of motor performance in children, have the potential to greatly improve the QOL of their caregivers, particularly in the physical domains. According to the guideline for rehabilitation of CP published by the *Japanese Association of Rehabilitation Medicine* in 2014 (31) and recent reviews (3, 32), currently, there are only a limited number of randomized studies with significant findings on the therapeutic efficacy of hippotherapy with respect to gross motor function of the study participants over a short-term evaluation period (usually consisted of a 12-weeks intervention). However, previous research findings were diverse due to the mixed levels of gross motor functions of the study participants, length of the training period, heterogeneous etiology and various outcomes investigated (33). This is the first prospective study to examine the benefits of long-term hippotherapy on the gait function and related gait parameters in children with CP. The present results also indicate that hippotherapy may have important ancillary effects to improve the psychological QOL of their primary caregivers.

It is known that gait analysis is useful for evaluating the effect of CP on lower body mobility (34). Our findings are in line with the hypotheses that three-dimensional gait analysis using a portable motion recorder could be a sensitive biomarker to predict the 1-year outcomes of gross motor function domains following hippotherapy (19). Notably, in this study, improved quality of gait function after long-term hippotherapy was estimated successfully by comparing the relationships of select quantitative parameters (gait acceleration or center of mass displacement vs. cadence) in comparison to age-matched healthy subjects. These results support the finding that hippotherapy can significantly improve the quality of gait through stabilization of dynamic balance and functional performance, including trunk stability (22).

At present, no data is available with regard to the motor outcomes of participants after completion of the hippotherapy program. In this study, we prospectively followed children with CP for 3 months after their 1-year hippotherapy experience. The 3-month follow-up duration was chosen because we found that the preferable effects of long-term hippotherapy on walking and balance abilities usually persisted for about 3 months, and during that time, we had to make a critical decision regarding whether to start another session in order to prevent possible deterioration (Mutoh and Tsubone, unpublished observation). In addition, there are some cases of adolescents and young adults with CP that may have a potential for further improvements in some gait parameters with weekly hippotherapy extending to over 1.5–2 years (18), which supports the benefits of continuous, or at least long-term, training programs.

Hippotherapy provided a significant improvement in the psychological domain scores of the WHOQOL-BREF instrument across the two follow-up time points. Interestingly, this result validated the suspicion of a positive relationship between the changes in the psychological QOL, particularly in the “positive feeling,” of caregivers and improvement in the children's gait functions after hippotherapy. Conversely, several reports have shown that the health-related QOL of caregivers is not necessarily associated with their children's level of functioning (35) or with improvements in their level of performance (23). Although the sample size in our study was small, which may have restricted the interpretation of our results, it is conceivable that hippotherapy may provide better psychological QOL within the family caregivers of children with CP by recognizing their children's gait function improvements through successful horseback-riding sessions. Further studies using substantially larger sample sizes are warranted to determine the longitudinal impact of a hippotherapy program on the psychological outcomes of caregivers in more detailed, as measured by factors such as caregiver burden; QOL; anxiety; depression; perceived control; stress mastery; and caregiver confidence, preparedness, and mastery.

This study has some limitations that may concern its readers. Although we used similar sample sizes in both groups with a more rigorous methodology, the study does not convincingly confer further advantage to those of other past smaller sample size studies; so, our results need to be viewed with caution. Our study was not double blinded in design, the sample size was small, and the follow-up duration may have been insufficient. Due to the limited range of participant characteristics and resources at the facilities, we could not enroll more children or follow-up with them for longer than 3 months after the program. Indeed, as a result of the knowledge and experience on the benefits of hippotherapy (3, 36), most caregivers in both groups were adamant about assigning their children to the next intervention as early as possible after the completion of this study. Future investigations that focus on follow-up protocols to achieve better outcomes may be needed.

## Conclusion

The present data indicate that a 1-year program of once-weekly hippotherapy provides an improvement in the walking ability of children with CP and increases the QOL of their caregivers, suggesting the substantial advantage of utilizing this type of training on a long-term basis. In Japan, clinical evidence of animal-assisted therapy for children with CP was poorly available, compared with that in other countries, because of less commitment of rehabilitation physicians/therapists to this field (usually directed by support staff and veterinarian in the horseback riding center). Present methodologies can benefit future studies that focus on understanding hippotherapy for children with CP underlying gait impairment and caregivers' psychosocial problems that facilitate the efficacy of potential treatments.

## Data Availability

All datasets generated for this study are included in the manuscript and/or the Supplementary Files.

## Ethics Statement

This study was approved by the Human Research Ethics Committee of the Society of Physical Therapy Science (SPTS2016007). The trial was registered in the University hospital Medical Information Network Clinical Trials Registry (UMIN-CTR) in July 2, 2016 (UMIN000022986).

## Author Contributions

ToM and TaM co-designed the study. MD, MasayI, and MasahI recruited subjects and assisted with the intervention. MT and HS analyzed the gait signals. TaM and HT proposed the research topic. ToM and TaM organized data collection, signal processing, and manuscript draft. YT and MasahI supervised the study. All authors contributed to the interpretation of results as well as in the writing and critical review of the manuscript. All authors have approved the final version of the manuscript.

### Conflict of Interest Statement

The authors declare that the research was conducted in the absence of any commercial or financial relationships that could be construed as a potential conflict of interest.
