# Supplementary material for: Impact of Long-Term Hippotherapy on the Walking Ability of Children With Cerebral Palsy and Quality of Life of Their Caregivers
Source: Front Neurol. 2019 Aug 13;10:834. doi: 10.3389/fneur.2019.00834 (PMC6701452; doi:10.3389/fneur.2019.00834)
Supplement: Supplementary file 1 [file Data_Sheet_1.doc]

**Separate Document Detailing the Trial Protocol**

**Ethics and Registration**

This study was conducted in compliance with the ethical guidelines of the Declaration of Helsinki and approved by the Human Research Ethics Committee of the Society of Physical Therapy Science (SPTS2016007). All experiments in this study were performed in accordance with the relevant guidelines and regulations, and written informed consent was obtained from the caregivers of all subjects. The trial was registered in the University hospital Medical Information Network Clinical Trials Registry (UMIN-CTR) on 2 July 2016 (UMIN000022986).

**Participants and Setting**

All eligible participants were recruited and tested from July 2016 to March 2018. Children with CP were recruited prospectively by contacting the schools that cater to such children by providing appropriate physiotherapy services and from adjacent university and/or other general hospitals. A total of 30 participants were enrolled. All participants had 1) the ability to walk with or without aids or braces, 2) a prior diagnosis of spastic CP, and 3) an ability to train for more than 30 min. There were four exclusion criteria: 1) having had any orthopaedic surgery or other surgical interventions that may have influenced mobility in the past 6 months, 2) having received a botulinum toxin injection in the past 3 months or a selective dorsal rhizotomy in the past 1 year, 3) impairments that could contraindicate horseback riding, and 4) other medical conditions that could materially influence mobility. No participants had any previous experience with hippotherapy.

**Study Design**

This study was designed as a randomised controlled trial. Participants were allocated by computer-generated stratified randomisation according to the severity of their impairment (assessed by the Gross Motor Function Classification System [GMFCS]: mild, I−II; moderate, III, and severe, IV−V) to one of the following two treatment groups: outdoor recreation (control) and hippotherapy. BellCurve for Excel software (SSRI, Tokyo, Japan) was used to calculate the appropriate sample size. Based on the results of a previous cohort study , 11 children in each group were needed to detect a mean change of 3% in the Gross Motor Function Measure (GMFM)-66 scores in order to benefit from hippotherapy (with the assumption of no apparent change detected in the control group receiving conventional physiotherapy) with a power of 80% and type-I error rate of 0.05.

**Training Protocol**

Hippotherapy was performed at the Holistic Betterment and Wellness Through Riding PIROUETTE riding centre (http://www.pirouet-u.com/) formerly known as Riding for the Disabled Association (RDA) Utsunomiya (Tochigi Prefecture), located in the Kanto region–Japan’s largest plain consisting of seven prefectures including the capital city of Tokyo (https://www.japan.go.jp/regions/kanto.html). The facility has licensed and specially trained members including therapeutic riding instructors, occupational therapists, psychologists, medical doctors, and veterinarians and conducts highly dedicated educational and research activities on hippotherapy . The facility holds 3 Thoroughbreds, 2 Welsh ponies, 2 Hokkaido native horses, 1 Arabian, and 1 Haflinger trained by the staff to participate in the program.

Participants underwent a 30-min program of hippotherapy once a week for 48 consecutive weeks (1 year) in an outdoor riding arena. Horses and participants were matched according to the size and functional status of the children with the size and movement characteristics of specific horses, as best as possible. Certificated therapeutic riding instructors from RDA (Riding for the Disabled Association) Japan (http://rdajapan.or.jp/) and Australia (http://www.rda.org.au) planed and performed a professional and safe hippotherapy treatment session which requires: matching the horse and the child, positioning the child on the horse as well as applying all necessary adjustments including using various aids, defining the duration of hippotherapy treatment session and its intensity, as well as selecting the appropriate terrain. The hippotherapy used in this study was based on the PIROUETTE program , which included muscle relaxation and sustenance of optimal postural alignment of the head, trunk and lower extremities with independent sitting and active exercises (stretching, strengthening, dynamic balance and postural control), as directed by the instructor (**Figure S1A**).


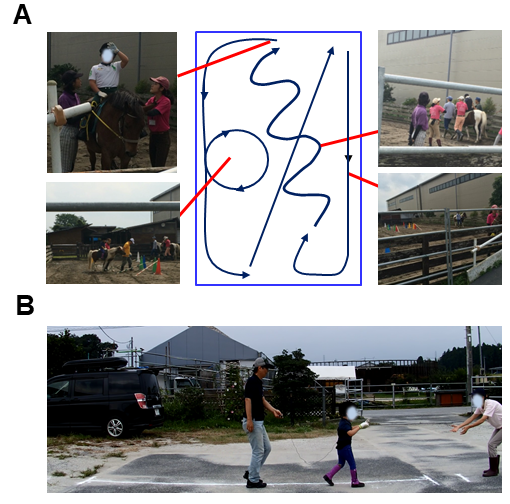


**Figure S1. (A)** Riding arena. The horse was led to a walk around an arena (27 m × 12 m) in clockwise and counter-clockwise directions, including a winding ride and slalom. The sessions were conducted by a therapeutic riding instructor accredited by the Riding for the Disabled Association Japan, and the horse was led by a trained assistant. Further, three staff members, including one who led the horse at the walk and two who walked at the horse’s side to help prevent a fall, ensured that the protocol was followed and that it was safe, but they did not provide postural support to the participant at any time. The participant was placed on a saddle wearing a helmet and was asked to actively adjust their posture. **(B)** The experimental setup for the 5-m walk test. Each participant had an attached portable motion recorder with a belt placed around the hip and walked on a 7-m flat walkway, where the walk in the middle 5 m of the walkway was timed by an examiner who walked behind the participant.

In contrast, participants assigned to the control group received a weekly recreation program using outdoor amenities such as green spaces, walking path, and playground equipment using the same time schedule as the hippotherapy cohort (i.e., a 1-year weekly program followed by a 3-month follow-up). All participants continued their normal daily routines for the rest of the week, but none of them received routine physical therapy during the study period.

In both groups, caregivers were allowed to watch the session without participating in the protocol or discussing the contents or answers of the self-reported questionnaire form with each other so as to avoid bias via the formation of alliances with other caregivers during the session, which could have contributed to a feeling of psychological well-being, especially in the hippotherapy caregiver group.

**Clinical Assessments**

Physical function was assessed by a 5-m walk test (5MWT) before and after each session at self-selected walking speeds . The 5MWT was performed on a 7-m walkway, and the walk in the middle 5 m of the walkway was timed. Participants who habitually wore ankle–foot orthoses and/or used a walking aid were permitted to use them for the test (**Figure S1B**). Temporospatial analyses of the gait and balance abilities were performed using a portable motion recorder (MG-M1110-HW; LSI Medience, Tokyo, Japan) (**Figure S2A**). The resulting motion signals were then recorded at a sampling rate of 100 Hz, stored on a computer for off-line analysis (MG-M1110-PC; LSI Medience, Tokyo, Japan) (**Figure S2B**) and then used to quantify cadence (step/min), walking speed (m/min), step length (cm), average acceleration (G), and the horizontal (side-to-side motion of the pelvis) and vertical (rise and fall of the centre of gravity) displacement ratio (cm) .

The overall QOL of caregivers was investigated using the brief version of the World Health Organization Quality Of Life (WHOQOL-BREF) self-assessment questionnaire derived from the WHOQOL-100 . It contains 26 universal items that address five domains of QOL: general health (two items), physical health (seven items), psychological health (six items), social relationships (three items), and environment (eight items) (<https://www.who.int/healthinfo/survey/WHOQOL_BREF.pdf?ua=1>). These domains and terms were agreed upon by the international consensus and adapted into a Japanese version . In this study, the WHOQOL-BREF was self-administered since all of the respondents were considered to have sufficient ability to complete the form, as described . The domain scores were scaled in a positive direction ranging from 1–5 points, with higher scores denoting better QOL.


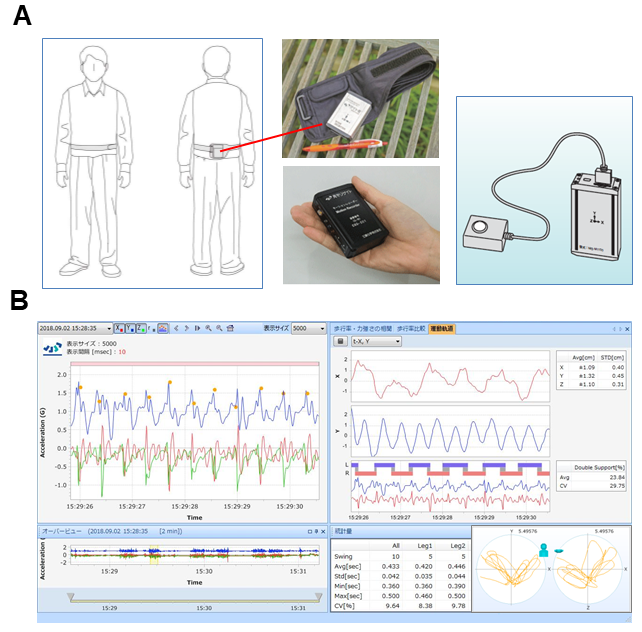


**Figure S2.** **(A)** A portable motion recorder with a wired remote-control device is fixed using a belt placed around the hip parallel to the lumber spine. **(B)** Original tri-axial acceleration data (upper panels) and centre of mass path (right lower panels) in the coronal (*X-Y*) and axial (*X-Z*) planes across the range of walking speeds.

**Outcome Measures**

Temporospatial gait parameters and QOL domains of the caregivers were compared between the two groups before (baseline) and after the 1-year intervention and at the follow-up 3 months after the completion of the training (post-training). GMFM-66 and GMFM dimension-E (GMFM-E) (walking, running, and jumping) scores of GMFM were measured for outcome analyses by the same blinded examiner. Each measurement was performed under the same conditions for a total of three times: the first one was made the week before beginning the intervention, the second one the week after the completion of the 1-year intervention, and the final one on the day of the 3-month follow-up. Due to the limited range of participant characteristics and resources at the facilities, we could not enrol more children or follow-up with them for longer than 3 months after the program. Indeed, as a result of the knowledge and experience on the benefits of hippotherapy , most caregivers in both groups were adamant about assigning their children to the next intervention as early as possible after the completion of this study.

**Statistical Analysis**

Continuous data that were normally distributed according to the D’Agostino–Pearson normality test were compared using analysis of variance with *post hoc* Bonferroni–Dunn correction. Non-normally distributed variables were assessed using the Mann–Whitney *U* test. Univariate analyses of the relationships between categorical variables and outcomes of interest were performed using the χ^2^ test or Cochran–Armitage test as appropriate. Linear regression analysis was used to compare the average acceleration or horizontal/vertical displacement ratio with cadence (potential predictors of gait and balance stabilities for long-term hippotherapy) for within-group correlation both before and after the intervention. Multinomial logistic regressions were fitted to determine if data plots for each group were associated with the referenced NDB values predefined using age-matched healthy subjects (**Figure S3**). All analyses were performed using SPSS (version 24.0; IBM, Armonk, NY, USA) and Prism (version 8; GraphPad Software, La Jolla, CA, USA). Data are expressed as mean ± standard deviation (SD). The level of significance was set at *p* < 0.05.


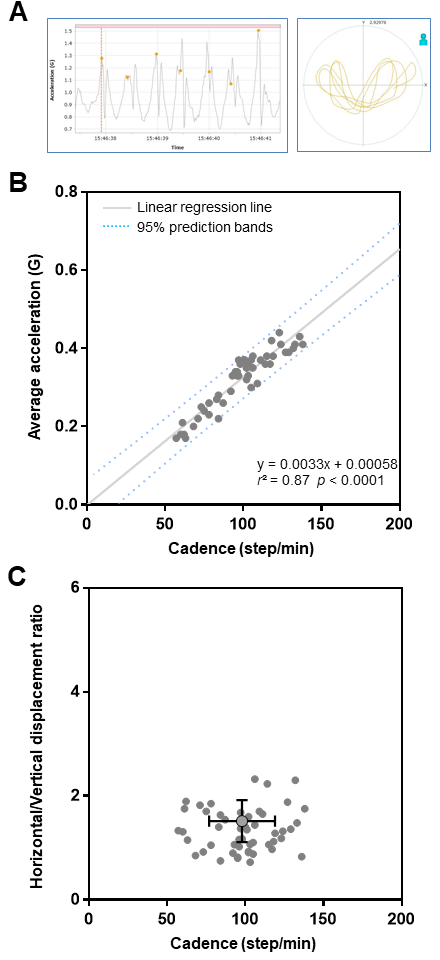


**Figure S3.** **(A)** An example of the centre of mass path in the coronal (*X-Y*) plane (right panel) across the range of walking speeds in a 5-m walk test (left panel). **(B)** Linear regression analysis of cadence and average acceleration in 50 age-matched normal subjects (25 males and 25 females; age, 8.5 ± 2.9 years) showed a positive relationship; dashed lines indicate 95% prediction intervals of the regression line. **(C)** Scatter plot showing the relationship between horizontal/vertical displacement ratio and cadence; mean plots with both horizontal and vertical standard deviations are also superimposed. The horizontal/vertical displacement ratio was normally distributed (F distribution 0.95; degree of freedom 50; *p* = 0.03) within a narrow range (mean = 1.34; SD = 0.41; ranging from 0.73 to 2.32). However, there was no relationship between the balance and gait strength parameters (*r*^2^ = 0.0049; *p* = 0.63).

**References**

1. Kwon JY, Chang HJ, Lee JY, Ha Y, Lee PK, and Kim YH. Effects of hippotherapy on gait parameters in children with bilateral spastic cerebral palsy. *Arch Phys Med Rehabil.* (2011) 92:774-9. doi: 10.1016/j.apmr.2010.11.031

2. Ihara M, Ihara M, and Doumura M. Effect of therapeutic riding on functional scoliosis as observed by roentgenography. *Pediatr Int.* (2012) 54:160-2. doi: 10.1111/j.1442-200X.2011.03456.x

3. Mutoh T, Mutoh T, Takada M, Doumura M, Ihara M, Taki Y, et al. Application of a tri-axial accelerometry-based portable motion recorder for the quantitative assessment of hippotherapy in children and adolescents with cerebral palsy. *J Phys Ther Sci.* (2016) 28:2970-4. doi: 10.1589/jpts.28.2970

4. Mutoh T, Mutoh T, Tsubone H, Takada M, Doumura M, Ihara M, et al. Impact of serial gait analyses on long-term outcome of hippotherapy in children and adolescents with cerebral palsy. *Complement Ther Clin Pract.* (2018) 30:19-23. doi: 10.1016/j.ctcp.2017.11.003

5. Mutoh T, Mutoh T, Tsubone H, Takada M, Doumura M, Ihara M, et al. Effect of hippotherapy on gait symmetry in children with cerebral palsy: A pilot study. *Clin Exp Pharmacol Physiol.* (2019) 46:506-9. doi: 10.1111/1440-1681.13076

6. McGibbon NH, Andrade CK, Widener G, and Cintas HL. Effect of an equine-movement therapy program on gait, energy expenditure, and motor function in children with spastic cerebral palsy: a pilot study. *Dev Med Child Neurol.* (1998) 40:754-62.

7. Wilson CM, Kostsuca SR, and Boura JA. Utilization of a 5-meter walk test in evaluating self-selected gait speed during preoperative screening of patients scheduled for cardiac surgery. *Cardiopulm Phys Ther J.* (2013) 24:36-43.

8. Skevington SM, Lotfy M, and O'Connell KA. The World Health Organization's WHOQOL-BREF quality of life assessment: psychometric properties and results of the international field trial. A report from the WHOQOL group. *Qual Life Res.* (2004) 13:299-310. doi: 10.1023/b:Qure.0000018486.91360.00

9. Tazaki M, and Nakae Y. *WHO QOL 26 Japanese Version.* Tokyo: KANEKOSHOBO (1997).

10. World Health Organization. *WHOQOL: Measuring Quality of Life.* 3. Administration of the WHOQOL instruments. (2019) [Online]. Available at: https://www.who.int/healthinfo/survey/whoqol-qualityoflife/en/index2.html (Accessed May 25, 2019).

11. Koca TT, and Ataseven H. What is hippotherapy? The indications and effectiveness of hippotherapy. *North Clin Istanb.* (2015) 2:247-52. doi: 10.14744/nci.2016.71601

12. Martin-Valero R, Vega-Ballon J, and Perez-Cabezas V. Benefits of hippotherapy in children with cerebral palsy: A narrative review. *Eur J Paediatr Neurol.* (2018) 22:1150-60. doi: 10.1016/j.ejpn.2018.07.002
